# An Anonymous Channel Categorization Scheme of Edge Nodes to Detect Jamming Attacks in Wireless Sensor Networks

**DOI:** 10.3390/s20082311

**Published:** 2020-04-18

**Authors:** Muhammad Adil, Mohammed Amin Almaiah, Alhuseen Omar Alsayed, Omar Almomani

**Affiliations:** 1 Department of Computer Science, Virtual University of Pakistan, 54–Lawrence Road, Lahore 54000, Pakistan; 2 Department of Computer Science, King Faisal University, Al-Ahsa 31982, Saudi Arabia; malmaiah@kfu.edu.sa; 3 Deanship of Scientific Research, King Abdul Aziz University, Jeddah 21589, Saudi Arabia; aoalsayd@kau.edu.sa; 4 Computer Network and Information Systems Department, The World Islamic Sciences and Education University, Amman 11947, Jordan; Omar.almomani@wise.edu.jo

**Keywords:** WSNs, frequency division multiplexing, security, jamming attacks, edge nodes, authentication, bandwidth

## Abstract

Wireless Sensor Networks (WSNs) are vulnerable to various security threats. One of the most common types of vulnerability threat is the jamming attack, where the attacker uses the same frequency signals to jam the network transmission. In this paper, an edge node scheme is proposed to address the issue of jamming attack in WSNs. Three edge nodes are used in the deployed area of WSN, which have different transmission frequencies in the same bandwidth. The different transmission frequencies and Round Trip Time (RTT) of transmitting signal makes it possible to identify the jamming attack channel in WSNs transmission media. If an attacker jams one of the transmission channels, then the other two edge nodes verify the media serviceability by means of transmitting information from the same deployed WSNs. Furthermore, the RTT of the adjacent channel is also disturbed from its defined interval of time, due to high frequency interference in the adjacent channels, which is the indication of a jamming attack in the network. The simulation result was found to be quite consistent during analysis by jamming the frequency channel of each edge node in a step-wise process. The detection rate of jamming attacks was about 94% for our proposed model, which was far better than existing schemes. Moreover, statistical analyses were undertaken for field-proven schemes, and were found to be quite convincing compared with the existing schemes, with an average of 6% improvement.

## 1. Introduction

Advancement in electronics and wireless communication systems leads to the implementation of wireless sensor networks (WSNs). WSN is a collection of wireless nodes, which are deployed in unstructured environment where they can collect and process information according to their assigned task. WSN contains several wireless nodes, which has storage, processing and communication capabilities. Wireless sensors are tiny devices, which are deployed in an infrastructure-free environment to collect information according to their assigned task [1]. Moreover, these networks have various applications in the real world. In the recent past, wireless sensor network applications have been extended from general to specific uses with the passage of time. The specific application of WSNs includes emergency response systems, weather forecasting, agriculture, body area network and security assessment systems.

The cost of wireless nodes varies according to their size, on board battery charge, processing, computing, receiving and transmitting capabilities. A normal wireless sensor network consists of hundreds to thousands of sensor devices. They work according to their assigned task in the deploying area. The structure (topology) of these networks can be categorized according to their deployment environment [2]. WSNs are mainly categorized into three types, which includes cluster based, flat based and hierarchical based networks. The deployment of sensor devices in inaccessible, harsh and remote locations increases its importance, because these devices have limited storage, power, bandwidth and communication capabilities. Similarly, open and shared transmission medium of these network increases its susceptibilities to different kind of transmission attacks.

Different types of WSN attacks that affect the integrity, authenticity and availability of information includes denial of service attacks (DoS), jamming attack, black hole attack, eavesdropping attacks, distributed denial of service attacks (DDoS), sybil attack and wormhole attack. Authentication of operational devices in WSNs is one of the challenging task to the research community, because of the self-organizing and dynamic nature of these devices. Moreover, due to the constraint oriented behavior of these nodes, some generalized authentication schemes exists in the literature to develop a secure communication infrastructure for these networks. Likewise, the MAC-AODV scheme is proposed in article [3] to maintain the physical and network layer security of constraint oriented networks. However, this scheme identifies the malicious nodes in the network by means of MAC address matching in the base station.

There are also some routing, data eavesdropping and Man-in-Middle attacks in WSNs, where the attacker uses the transmission medium to capture network traffic and hijack networks security. Similarly, jamming attack is also a type of transmission attack, where the attacker uses a high range of transmission signals with the same frequency transmissions to disrupt the network security. However, jamming attacks are also happening, due to some unintentionally wireless medium interferences such as noise and collision etc. [4]. Moreover, jamming attacks in wireless sensor networks constitutes the physical interference of an attacker to disturb the communication process. The objective of DoS attacks in a wireless network is to direct an unwanted transmission signal toward the sensor node to disrupt communication channel, bandwidth, battery resources, storage resources and transmission line of destination node. Furthermore, this not only disturbs the communication process, but also minimizes the network lifespan [5].

Jamming attack in wireless sensor networks is one of the simplest types of attack that an attacker uses to disrupt network security, because it does not need any special type of hardware or software. To elaborate upon the concept of jamming attacks in a precise way, this attack can be managed by listening to the transmission medium passively to identify the transmission frequency and generate the same frequency signal to disrupt the legitimate communication process. Likewise, a common jamming attack is characterized by a low detection rate, high energy efficiency and anti jamming resistance [6]. Jamming attack detection has been an ongoing research area in wireless networks over the last decade. The researches community proposed various scheme to address the issue of jamming attacks in WSNs. However, in most of the cases these techniques are specific to operation, software, hardware OR environment.

Moreover, the research community also worked on the prior communication information (behavior of transmission) of the wireless networks to identify jamming attacks. Furthermore, they used indicators and trackers to verify the existence of jamming attacks in WSNs [7]. The example of these behaviors includes signal strength of transmitting packet at the physical layer and packet lost ratio at the 7th layer of the OSI model (application layer). The cross layer model (CLM) scheme was used to combat the collection of jamming attacks in wireless networks with the help of an indicator [8]. A multi model scheme for detection of jamming attacks in 802.11b wireless networks is proposed in [9]. In this model, they used the correlation parameters such as signal strength variation, packet delivery ratio and width of the received signal to identify jamming attacks in deploying WSNs. Zhang et al. [10] proposed the optimal attack scheduling technique to investigate jamming attacks in WSNs. He used a remote state estimator to check the reliability metrics of data at remote end of deployed WSN. However, this scheme was affective for a specific area of WSNs, where the attacker block individual nodes transmission channels. In case, if the attacker generate a high interference frequency in the transmission channel, then the proposed model is not effective acknowledge the existence of jamming attack in WSNs. Moreover, in the proposed model, the jamming attacks detection rate was based on information such as covariance error of communication, for which is not possible to be consistent every time, due to the dynamic nature of wireless nodes in WSNs. Therefore, the performance reliability metrics for covariance error do not remain constant in a deployed WSN environment.

The optimal jamming attack detection scheme was proposed by Li et al. [11]. In the proposed scheme, he used monitor nodes to detect estimated jamming attacks in the deployed WSNs. However, wireless sensor networks are deployed in an inaccessible, remote and harsh location, where monitoring of monitor nodes is not possible. Therefore, the proposed model is only effective for small accessible regional based WSNs. Pradhan and Venkitasubramaniam proposed the K-L divergence scheme for detection of dynamic data injection in WSNs [12]. In dynamic data injection, the attacker gets physical access to the transmission medium and modify the sequence of temporal data in the network. The proposed model uses the K-L divergence mechanism to identify dynamic data injection attacks in WSNs by means of comparing the values of prior and posterior-distribution of arriving data at remote location.

The existing techniques of jamming attacks are mostly based on the single channel and single sensor node transmission channels. However, jamming attacks under multiple channel and sensor nodes are an interesting issue for research community to detect and prevent it accurately in operational networks. By the encouragement of this statement, we considered a wireless network infrastructure (topology) of dynamic number of nodes in this research, whose communication is controlled by three edge nodes and round trip time (RTT). RTT is the amount of time that messages need to travel from source to destination in communication infrastructure. The edge nodes first run the embedded algorithm to calculate the estimated value of RTT at the local side (edge node). likewise, the edge node transmits the message packet, which contain information of estimated RTT of local site to a remote location by means of communication channel. At a remote destination, the estimator calculates the expected value of RTT for the incoming message packet. Similarly, the remote estimator compares both values of RTT (source and destination) to maintain the performance reliability of communication in the network.

Moreover, the three edge nodes used in the deployed area of wireless sensor network have different transmission frequency channels in the same bandwidth. All the three edge nodes calculate the estimated RTT of a packet before transmitting it to the transmission media and the estimated value of RTT is sent in the payload of the packet, where at remote location it is compared to estimator calculated value of RTT to maintain transmission reliability. Similarly, if an adversary wants to disrupt the communication process or degrade the network performance the adversary generates the same frequency signal as that of the edge node transmitting signals to disturb the communication process. In this case, the adversary can only disturb the communication process of only one edge node, because all three edge nodes have different communication channels based on embedded algorithm configuration. Keep in mind the existing schemes of jamming attacks, which verifies that the attacker can jam only one channel at a time to disrupt the communication process. The three edge nodes and channel segregation in our proposed scheme address the aforementioned issue of jamming attacks in WSNs with simple network implementation. Moreover, the proposed model creates a secure infrastructure for deployed WSNs, where they can collect and process information according to their assigned task. Similarly, the secure communication environment maximizes network performance by accurately detecting jamming attack, low latency and high throughput.

### 1.1. Problem Statement

Detection and prevention of jamming attack in WSNs through an anonymous edge node scheme.

There exists a WSN, which contains a dynamic number of sensor nodes (S), where *S* = ($S_{1}$, $S_{2}$, $S_{3}$–$S_{n}$). The *S* nodes collects information and transmit it to a remote destination through edge nodes ($E_{i}$), where $E_{i}$ = ($E_{1}$, $E_{2}$ and $E_{3}$). The $E_{i}$ nodes has different frequency channels (CH), where CH = ($CH_{1}$, $CH_{2}$, and $CH_{3}$).

### 1.2. Objective of Research

1. Design a secure communication infrastructure for deploying wireless nodes (S), where *S*∈$S_{n}$ for designated WSN.
2. Deploy $E_{i}$ nodes in designated WSNs.
3. Categorise the Transmission channels (CH).
4. Ensure transmission channel security.
5. Detect jamming attacks
6. Claim justified results for our scheme in a simulation environment.

The rest of the paper is organized as follows. The subsequent section of the paper overviews the related work of jamming attacks. Section 3 of the paper contains the proposed methodology with adopted algorithm of our scheme. Section 4 discusses the simulation experiment and result statistics of our scheme. The subsequent section concludes the paper.

## 2. Literature Review

In WSNs, the security of wireless devices and communication infrastructure is one of the most challenging issues for researchers, due to its dynamic and unpredictable nature. The literature suggests various schemes to resolve the issues associated with susceptibility and security of the WSNs. Many researchers worked on this issue to develop a secure communication infrastructure. Every network threat has their own aim to hijack the network security by means of monitoring network traffic, damage some wireless nodes, jam network traffic or disrupt the entire network. However, the most destructive attack among all of them is jamming attack, because it disturbs the entire communication of the network. Some of the suggested techniques for prevention of jamming attacks are mentioned below.

The multi joint relay and jammer selection scheme was proposed by Zhang et al. [13]. They used a secure decoder in their model to assess all incoming signal before and after transmission. Additionally, they used an artificial broadcast signal in their scheme to misguide the adversary/attacker, before launching attack on the communication channel. Feng et al. [14] used the random Markov chain process in their research to develop a multi agent system infrastructure for prevention of strategic cyber attacks in wireless networks. In this scheme, a hybrid stochastic secure control framework was used to investigate and identify jamming attack. However, after each and every investigation check, the connectivity restoration mechanism was mandatory, which increase the complexity and network overhead in designated WSNs. Tang et al. [15], suggested the triggering strategy model for detection of jamming attacks such as denial of service (DoS) in mobile robots. They check the proposed model in the operational environment of tracking control mobile robots to detect and identify jamming attacks in the form of DoS attacks.

Sharma et al. [16], proposed the lightweight behavior rule specification method to identify cyber physical attacks in WSNs. The proposed technique uses the communication behavior information of deployed sensor nodes to identify region based jamming attacks in the networks. The key concept of the proposed model was to identify the malicious sensor node in the network based on transmission behavior. Moreover, in the proposed model, an automatic verification process was enabled to continuously check the operational network for jamming attacks. Intrusion detection system (IDS) has been used for a couple of decades to protect networks from external attacks. However, applying traditional and ordinary IDS to WSNs is very difficult, due to its limited resources and dynamic nature. Zarpelao et al. [17], overviews in their survey the utilization of IDS in WSNs and IoT. Furthermore, he specifically highlight the attacks, where IDS can play an important role in WSNs environment. The attacks that were highlighted in this paper include routing base attacks, IP spoofing attack, DoS attack and DDoS attacks.

The fault detection scheme for an unmanned surface vehicle system (USVS) was proposed by Ma  et al. [18]. The USVS model is based on three phases, which includes event triggering, event base switching and piecewise function to verify the legitimacy of the network traffic and transmission medium. The event based triggering schemes had great importance to detect and prevent jamming attacks in WSNs. Additionally, the USVS scheme uses information such as communication delay and disturbance in communication process to detect jamming attacks. Ge et al. [19], in their article briefly provided an overview of the existing techniques adopted for prevention of jamming attacks in WSNs. The new resilient based security (NRBS) scheme for prevention of jamming attacks was proposed by Shammari et al. [20]. In NRBS scheme, the authors used information such as data delivery and power consumption of participating ordinary nodes to detect estimated jamming attack in WSNs.

Lu et al. [21] proposed the channel training method to identify covert pilot spoofing attacks in WSNs. The channel training scheme was very effective at detecting eavesdropping and routing attacks in WSNs. However, the attack categorization were made on the basis of transmission channel threshold value, which can not be effective in the worst condition of transmission. Therefore, the channel training model was effective in a normal WSNs communication environment, but during external interferences such as weather, fidelity and attenuation, the effectiveness ratio of the proposed model was found to be degraded. Saeed et al. [22] suggest an intelligent security scheme, which is known as random neural networks (RNNs) to detect jamming attacks in WSNs. The RNN scheme uses an application source instruction to detect malicious activity in the network. Moreover, the tags of application are broadcasted in the proposed model to verify the authenticity and legitimacy of the network traffic.

The non-orthogonal multiple access (NOMA) and Hybrid automatic repeat request (HARQ) scheme was proposed by Xiang et al. [23]. The combination of NOMA and HARQ were used in the proposed model to enhance the security of deployed WSNs. Jing et al. [24] briefly provided an overview of the security issues associated with three layers of IoT such as perception, transport and application layer. Moreover, they also suggest possible solutions to address the aforementioned issues in the IoT communication infrastructure. The LAM-CIoT lightweight authentication scheme was proposed by Wazid et al. [25]. The proposed model was used in a cloud-based IoT infrastructure to ensure the security of a collected date from a sensor at a remote location by means of a one-way cryptographic hash function authentication process. Wazid et al. [26] proposed the secure key management and user authentication model, which was known as SAKA-FC for fog computing environment to develop a secure communication infrastructure. They used a one-way cryptographic hash function with addition of bitwise-OR (XOR) operation to verify the legitimacy of participating devices in the network. The limitations of the existing literature are briefly summarised in Table 1.

### Limitation of the Literature

WSNs are very sensitive, due to their limited resources and dynamic behavior. Therefore, efficient utilization of these networks increases their productivity and reliability. WSNs consists of hundreds and thousands of wireless nodes, which are susceptible to different kind of attacks, due to their unattended or inaccessible environment. Jamming attack is one of the most damaging attacks to these networks, because it disrupts the communication process of deployed WSNs. The literature suggest various techniques to mitigate jamming attacks on WSNs environment, but most of them are specific to environment, system and software. Some limitations of existing techniques are described below:

1. Some of the existing schemes in the literature are specific to the system or operation [15].
2. Most of the mentioned schemes are complex in their implementation [13,14].
3. Some of the mentioned schemes have a complex authentication process, which generates network overhead [13,16].
4. Some of the mentioned schemes are effective against network attacks, but they are degrading network performance by means packet lost ratio, throughput and latency, etc., [13,14].

The solution proposed in this paper is not only efficient in network performance and reliability, but it is also accurate in jamming attack detection and has simple operational deployment.

## 3. Proposed Methodology: Three Edge Node Scheme

WSNs are deployed in different environment such as sea, forest, agriculture, mountain and health-care etc., where in most of the cases human access is not possible. These networks consist of sensor nodes, which collects information from their surrounding according to their directed tasks. However, these networks are susceptible to various security threats, which can affect their performance. Therefore, the design of a secure communication infrastructure can maximize its effectiveness and performance. WSNs are vulnerable to different security threats such as wormhole attacks, black hole attacks, DoS attacks, DDoS attacks, Sybil attacks and Jamming attacks etc,. Jamming attack is one of the most lurid types of attack for these networks, because it disrupts the communication process of WSNs.

The literature has suggested various schemes to combat jamming attacks in WSNs, but most of them are specific to operation or system, which minimizes their effectiveness in real world deployment. In the presence of existing literature limitations, an efficient three edge nodes scheme is proposed in this research to address the aforementioned issue of jamming attacks in WSNs. The three edge nodes are deployed in designated WSNs infrastructure, where they share the collected information of ordinary nodes with remote destination. Moreover, each edge node has different transmission channels in the same bandwidth, which enables the detection of jamming attacks in the transmission media. Therefore, our proposed model is very effective at detecting jamming attacks in WNS with a great accuracy. Moreover, it also maintains high throughput, minimum end to end delay and least packet lost ratio in the operational network.

To elaborate upon the concept of the proposed model, in this research, three edge nodes are used in the deployed WSN to transmit the collected information of ordinary nodes deployed in WSN topology. All the edge nodes have different transmission channels in our scheme, which they are using to communicate with remote destination. Moreover, all edge nodes (in our case three) have embedded built-in configuration to calculate the estimated RTT of a message packet and transmit the calculated value of RTT in message payload to remote destination. At remote destination, once the message packet is received, the estimator again calculate the value of RTT. After calculation of RTT at a remote location, the source message estimated time and remote location RTT is compared to maintain the reliability of the network and high quality detection of malicious activity.

**Figure 1:**
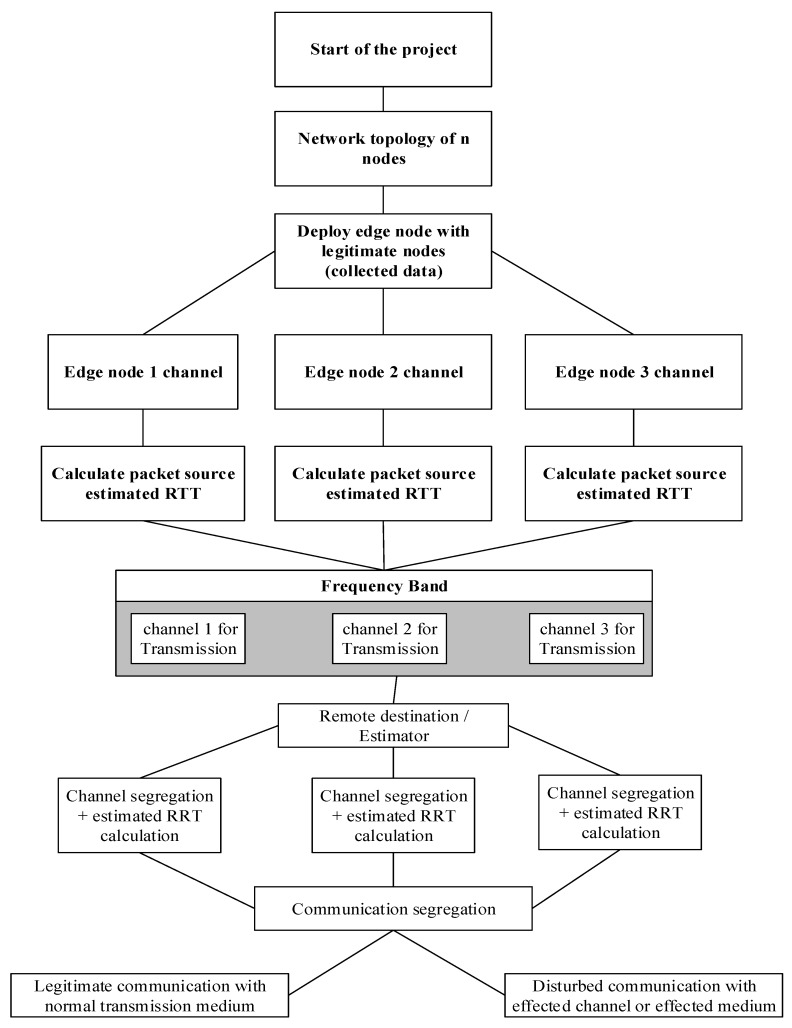
Step by step implementation process of our scheme in flowchart diagram.

To elaborate upon the concept of proposed model, the step by step process is shown in a flowchart diagram. Figure 1 of the paper shows the step by step implementation process of this research.

The above flowchart diagram of Figure 1 represents the complete overview of our proposed model. An ordinary network of a dynamic number of wireless nodes had been chosen with three edge nodes. Each edge node ($E_{i}$) with unique ID has a different frequency channel in the same frequency band. Likewise, the edge node transmits the collected information of deployed sensor network over the transmission media to a remote destination. However, each edge node has different transmission channel to transmit information from source to the destination location.

Additionally, each edge node at a time of transmission calculates the estimated RTT of every packet. Similarly, after reception of packets at a remote location, the estimated RTT is again calculated for each incoming packet. After calculation, the estimated RTTs are compared to verify the legitimacy of network traffic. However, the traffic legitimacy parameter is set for RTT with a threshold value of 15 μs. In the normal environment of WSNs, all incoming packet have less than 15 μs time RTT after comparison at a remote location. In case of interference in the communication channel, which we call malicious activity in the network, the value of RTT is increased from defined threshold value, which indicates the existence of external interference in the network. Similarly, if any incoming packet cross the threshold value of RTT, which is 15 μs, then this is the indication of a malicious activity in the network.

To elaborate upon the concept of RTT, in the ordinary communication condition, the RTT delay is less than 15 μs in our scheme. If, after comparison the RTT delay is more than 15 μs, then there is something wrong in the transmission media. Therefore, at remote locations, the same incoming frequency channel is kept under observation for 3 consecutive incoming packets. If all the incoming packets (3 consecutive packets) do not satisfy the estimated RTT condition of comparison (for source and destination RTT), an alarm message is broadcast, which is the indication of estimated jamming in the network. Moreover, every transmitted packet payload contain information such as ID, RTT, source and destination address, which make the communication process transparent in our scheme. Similarly, this is very helpful to identify the under attacks channel or edge node during operational network. Consequently, the communication of the channel under attack is completely unavailable at remote locations. In this case, the RTT importance is increased, because the channels adjacent to the channel under attack are also disturbed, due to high frequency interference, which disturbs the consistency of RTT during comparison. So the estimated RTT also ensures the existence of an estimated jamming attack in the adjacent channel. This is because the adjacent channels’ frequency is also effected with the attacking channel by means of high frequency interference. The RTT of the adjacent channels is calculated at the destination. The consistency of RTT will be found to be effected, which we have discussed in this paper.

**Figure 2:**
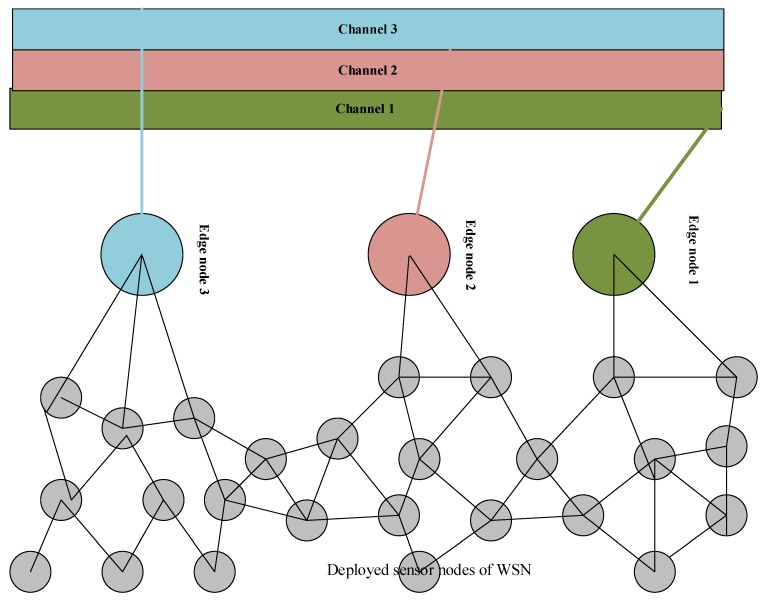
Channel categorization of edge node transmission for deployed WSNs.

Figure 2 of the paper represents the channel categorization of our scheme. The figure shows how the edge nodes collect information from deployed wireless nodes in the WSN infrastructure. Furthermore, the channel categorization is shown with different colors, according to edge node color, which explains the concept of our scheme. The edge nodes collects information form from deployed wireless nodes and forwards it to the specified transmission channel. Likewise, the specified channel transmits this information to remote locations. Moreover, the edge node ($E_{i}$) first calculates the estimated RTT.

In communication, RTT is very important to determine the accomplishment of connection. Moreover, in wireless networks RTT is profitable and efficient to measure retransmission time, window size and available bandwidth in transmission medium [27].

The RTT of a message packet can be calculated by the following Equations (1) and (2).

RTT=sourcequeuingtime+propagationdelaytime+destinationbuffertime

The above mentioned formula is used to calculate the RTT at edge node ($E_{i}$).

(1) RTT=α∗current−RTT(destination)−(α)∗newRTT(incomingpacket)

In the equation, α is the constant weight factor.

In Equation (1), the current RTT represents destination node, while the new RTT represents the new incoming packets. This is used for RTT comparison at remote sites. Moreover, to elaborate upon the concept of Equation (1), after reception of the message packet at remote destinations, the value of RTT is again calculated by following the same formula for each incoming packet. After calculation, the value of RTT is compared with source RTT value for each edge node. If they match, the communication process is continued in a smooth manner. However, if they did not match then the RTT value for an incoming packet is checked with time parameter, which is less than 15 μs for a legitimate incoming packet. If it exceeds the defined limit of 15 μs, then the incoming packets are consecutively seen for 3 RTT comparison. If the still do not match with the defined interval of time, then an alarm message is broadcast in the network to acknowledge the existence of tampering with the channel.

The RTT mainly depends on the sensor nodes’ presence in their round trip path and distance. The accuracy of RTT can be improved by reducing the number of sensor nodes present in the path of RTT. Our scheme is quite accurate by means of RTT calculation, because we have only two nodes involved in the communication process.

So the RTT for two sensor nodes is given by:

(2) $$\gamma *\;RTT=\gamma \;*\;E_{i}$$

In Equation (2), γ represents the delay from source node ($E_{i}$) and destination node ($D_{des}$). where $E_{i}$ = ($E_{1}$, $E_{2}$, $E_{3}$).

The edge nodes ε $E_{i}$ are almost at the same distance from the $E_{des}$ node.

As a result of equal distance, $\gamma_{1}$, $\gamma_{2}$ and $\gamma_{3}$ are all equal.

So γ = $\gamma_{1}$ = $\gamma_{2}$ = $\gamma_{3}$

All the $E_{i}$ have uniform RTT delay. Therefore, for the paired sensor nodes ($E_{i}$ & $D_{d}es$), RTT delay is obtained by Equation (3). Referring to Equation (2)

(3) γ∗RTT=3∗γ

This is the minimum RTT for a pair of sensors to exchange message packets in the operational WSNs. When under attack, the edge node ($E_{i}$) or channel can be identified by comparing the specific RTT values.

Let us assume that the communication channel is established among (($E_{i}$) and $D_{d}es$)), which is represented by (*M*) in the given Equation.

(4) C=N(N−m)

In Equation (4), C represents the number of channels used for communication. The analysis time required to calculate all $E_{i}$ nodes with RTT in the WSNs needs to be obtained. Therefore, the equation for analysis time of channel (C) is obtained and given by Equation (5).

(5) $$\gamma \;*Analysis\;\left(C\right)=\gamma \;*RTT_{1}\;+\gamma \;*RTT_{2}\;+\gamma *RTT_{3}\;+------\;\gamma \;*RTT_{c}$$

Referring to Equation (3), the optimal value of RTT for channel (C) is obtained by considering only two nodes ($E_{i}$ & $D_{des}$).

So, the obtained equation verifies the legitimacy, while calculating RTT for $E_{i}$ & $D_{des}$.

**Theorem** **1.** *An edge node $E_{i}$ exchange data with remote destination ($D_{des}$). **If**$E_{i}$ node RTT is <15 μs at $D_{des}$. **Accept**, **else**, observe and broadcast alarm.*

**Proof** **of** **Theorem** **1.** Let us suppose that the edge node $E_{i}$ calculates the estimated RTT time for the packet at the source end in the network. After calculating the RTT time, the $E_{i}$ node transmits the packet in the network through the specified frequency channel. After reception of the packet at destination ($D_{des}$), the estimator again calculates the estimated value of RTT for incoming packet. If the calculated value of RTT for the incoming packet matches the ($E_{i}$) RTT value, both the values are matched. □

Hence, the communication of $E_{i}$ is legitimate and the estimator at the destination allows the packet to be further processed.

**Conversely**, the $D_{i}$ RTT might not match the $E_{i}$ RTT time. Then, the estimator will deny the incoming packet and look for three packets to verify the legitimacy of the traffic.

Therefore, the afore-stated theorem verifies that only $E_{i}$ and $D_{des}$ matched estimated RTT values packets are allowed to communicate in the network.

Algorithm 1 of the paper comprehensively overviews the step by step process of the RRT time calculation of our proposed scheme, which is adopted for the calculation of legitimate traffic in the network. Let us assume that an Edge node $E_{i}$ initiates the communication process with a destination location/node. The $E_{i}$ calculates the RRT time of the packet before its transmission ($T_{x}$). Once the packet is transmitted through a communication channel, it contains all the necessary information such as source address, destination address and RRT time. Then the said packet is received at the destination location ($R_{x}$). The RRT time is again calculated and its value is matched with source packet RRT, if the time interval is <15 μs. Then the incoming packet allows for further communication in the network. If the incoming packet time interval is >15 μs, then 3 packets of the same source node are seen. If all three consecutive packets do not satisfy the defined interval of time (RRT) then an alarm message is broadcast on the network to acknowledge the existence of malicious activity.
**Algorithm 1** Legitimate traffic identification through RTT estimation**Require:** Allow only legitimate traffic, they have matched values of $E_{i}$ and $D_{i}$ RTT.**Ensure:**$E_{i}$ and $D_{i}$ RTT time matching. 1:$E_{i}$ initiate the process 2:$E_{i}$ Calculates ⟼ Estimated RTT at source side 3:$E_{i}$ compile ←packet 4:  $E_{i}$ transmit packet← through their frequency channel 5:  $D_{i}$ Estimator ← at remote location (recalculate RTT of $E_{i}$) 6:  $D_{i}$ matches RTT values of ←$E_{i}$
 7:   **If**
 8:   $D_{i}$ & $E_{i}$ RTT values matches ← Accept 9:   else10:   Checks for 3 consecutive packets11:   Deny after verification12:   $D_{i}$← allow network traffic of $E_{i}$
13:  **end if**
14:**return** Current information of specific $E_{i}$ node, traffic and channel.

### 3.1. Packets Lost Ratio in the Channel: Based on RTT

In the proposed model, the communication among sensor nodes and remote location (estimator) is through a wireless channel. We know that the unreliability of wireless medium means that there is more packet loss in the transmission process. Moreover, the packet loss process in this research process is defined through a random binary process $A_{k}^{i}$, where “A” is the RTT time. Likewise, in the proposed model, if the value of *i* = 1 at a remote destination (estimator), then the estimator or remote destination allows the traffic to be further processed. Conversely, if the value of *i* = 0, then the packets are lost in the communication process (channel) and the estimator looks for more incoming packets (at least 3 packets). Hence, the remote state estimator or destination fails to forward the incoming packets.

### 3.2. Jamming Attack: Based on RTT

Let us assume that an attacker wants to launch a jamming attack on the deployed network. The attacker has the capabilities to conduct the jamming attacks on the transmission media (transmission channel) and jam network traffic. However, the jamming of channel disrupts the communication process of edge node and remote destination/estimator, which causes dropout data packets in the process. The 3 edge nodes of the network send their data on different frequency channels when the attacker jams one frequency channel, because the other two channels are working. Likewise, the attacker is not blocking all channels at the same time in the network, so the other two channels help to identify the estimated jamming attack on the network. Moreover, the detection of jamming attacks is identified through the model shown below. A variable $J_{t}^{i}$ was chosen, variable *i* is the number of channels and t is the time. The channels are furthermore represented by the given statement as *i* = 1, 2, 3 and *t* = 0. As we discussed, only one channel, and in extreme cases two channels, can be blocked by an attacker at a given time. Keeping this in mind, when the attacker jams one or two channels in time *t*, then

(6) $$J_{t}^{i}=\;\;J_{t}^{3}\;=\;1-\;J_{t}^{3},\;\;J_{t}^{i}=1$$

where *i* = 1, 2, 3. likewise, when the attacker blocks two channels, then $J_{t}^{3}$ = 2, etc.

## 4. Experiment Results Analysis

The proposed scheme of jamming attack detection was implemented in OMNeT++. A real time simulation tool was designed for IoT and WSNs based projects. Moreover, the proposed model was developed by specifying a network area with the distribution of sensor nodes in an unstructured manner. The edge nodes were also distributed in the specified area of the network to transmit the collected information of sensor nodes to a remote location/destination. However, the edge nodes were configured with different frequency channels (transmission channels). By following Table 2 parameters, we connect all the sensor nodes and edge nodes with the suggested built-in configuration to achieve the required results and verify the performance metrics of our scheme compared with existing schemes. Furthermore, jamming attacks was launched against individual channels (each edge node transmission channel) to verify the detection rate of attacks and the RTT of adjacent channels in the operational network.

### 4.1. Formal Analysis of Proposed Scheme with It’s Rival Schemes

In this section, we have made a formal analysis of our proposed model compared with competitor schemes. WSN contains wireless nodes, which are very sensitive because of their limited resources such as memory, processing and communication. Moreover, these devices are vulnerable to different types of attacks. Jamming attack is one of the most destructive attacks to these networks. Feng et al. [14] used the random Markov chain process in their research to develop a multi agent system infrastructure for prevention of strategic cyber attacks in wireless networks. The limitations of the proposed model were its complex implantation and network overhead. Moreover, the connectivity restoration mechanism was mandatory in the propose model after each and every investigation, which creates an opportunity for an attacker to inject malicious data in the network. Similarly, during the investigation process, each and every wireless node participates, which consumes energy by means of transmissions and processing. The extra communication and processing in the proposed model minimizes WSN lifetime. Tang et al. [15], suggested the triggering strategy model for detection of jamming attacks such as denial of service (DoS) in mobile robots. The limitation of the proposed scheme was that it was specifically designed for mobile robots, which minimizes its use in the real deployment of WSNs. Sharma et al. [16] proposed the lightweight behavior rule specification method to identify cyber physical attacks in WSNs. The limitation of the lightweight behavior rule specification model was the network overhead and network lifetime because the proposed model uses a continuous verification process to identify jamming attacks in the network, which minimizes network performance. Moreover, the lightweight behavior rule specification model minimizes the lifespan of sensor nodes due to extra communication and processing, because these devices have limited battery power. In the real world deployment of WSNs, the behavior-based detection of jamming attacks is not particularly effective because there are various external interferences to a WSN transmission medium, such as fidelity and attenuation, which can change the transmission behavior of legitimate nodes of the network. Therefore, the lightweight behavior rule specification model is not very effective in a real WSNs environment. In this paper, we proposed a three edge node scheme to combat jamming attacks in WSN. The three edge nodes use three different channels for their transmission from the same deployed area. If an attacker blocks one channel in our proposed model, the communication process is not completely disrupted because the two other channels share information from the same deployed area, which verifies the serviceability of the network. Moreover, the advantage of our proposed model includes its simple implementation, high throughput, least end to end delay and accurate detection rate of the jamming attacks. Moreover, our proposed model does not need any special hardware or software in its implementation and operation phase. The results analysis observed for the proposed model based on throughput, end to end delay and jamming attacks were exceptional. Moreover, the simple implementation and communication process of the proposed model minimizes network overhead and maximizes network lifetime.

### 4.2. Performance Evaluation Based on Jamming Attacks Detection

The proposed model of our scheme was evaluated for jamming attacks. The attacks were launched on individual frequency channels to observe the detection statistics. Moreover, the attacks were also launched on the two frequency channels at the same time to overview the detection statistics. By launching jamming attacks in the said frequency channels, we generated the same frequency range in the network as that of legitimate channel frequency, bringing it in-line with the legitimate transmission channel to observe the jamming statistics. Then, the frequency interferences in the communication process were found to be disrupted in the remote destination/estimator. Likewise, the process is repeated for all three channels and the remote estimator/destination was observed for the resulting statistics. During simulation, the results observed for channels 1, 2, 3 are shown in the following graph. However, the overall statistics of our scheme were 94% while detecting jamming attacks from RTT estimator, which is far better than its rival scheme. The statistics of our scheme and its rival scheme are shown in the subsequent graphs.

### 4.3. Channel 1 Jamming Attacks Detection Statistics

**Figure 3:**
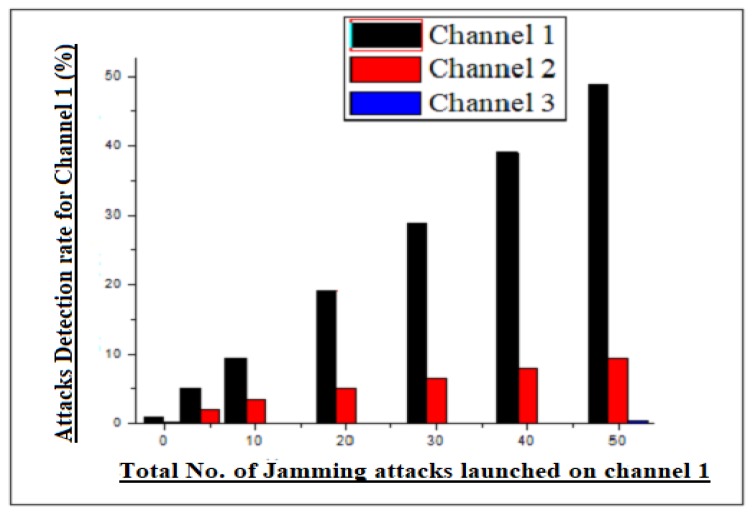
Channel 1 jamming attacks detection statistics.

As stated in the previous paragraph, the detection rate for the single channel jamming attack was found to be exceptional. The resulting statistics were verified at the remote estimator for channel 1. However, during the attack interval, the estimator at remote locations not only found the data dropout on channel 1, but it also checked the adjacent channel RTT time (Channel 2), which was found to disturb by means of consistency in the communication media. Moreover, the jamming attack detection statistic seen for channel 1 was quite accurate at remote locations, because channel 1 was completely dump. The RRT time of the adjacent channel, which was channel 2, was found to be disturbed, because in an ordinary environment, the data reception was quite consistent. The consistency of communication was found to be disturbed in channel 2 and 3, due to high frequency interference in the adjacent channel (channel 1). The graphical statistic observed during simulation, when channel 1 was under attack, is shown in Figure 3.

**Figure 4:**
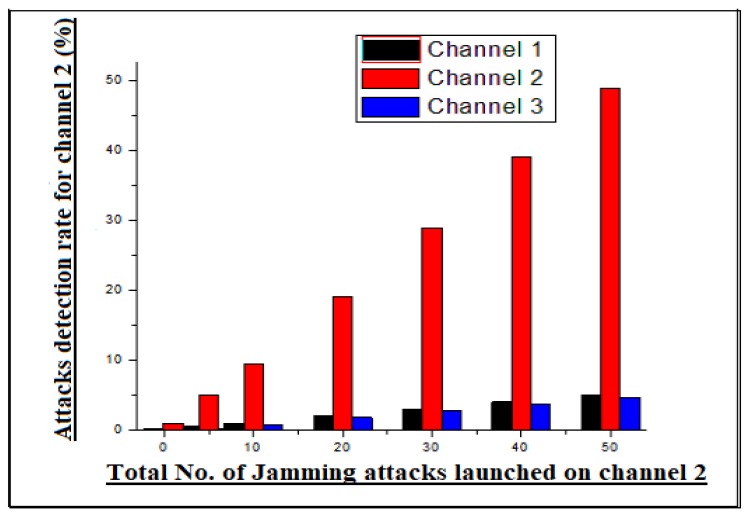
Channel 2 jamming attacks detection statistics.

**Figure 5:**
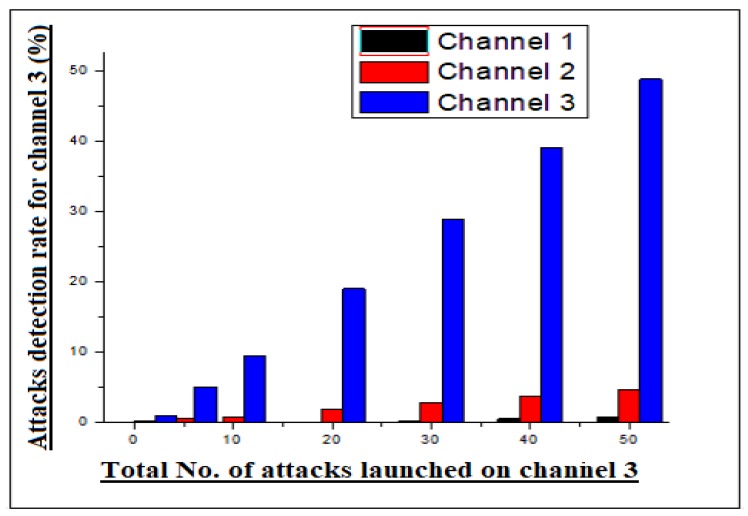
Channel 3 jamming attacks detection statistics.

In the same was as we tested channel 1, the results statistics were also calculated for channel 2. To verify the reliability metrics of our scheme, a jamming attack was also launched on channel 2. The communication of the channel was disrupted by means of generating high frequency signals against channel 2 transmission frequency. By generating a high frequency transmission signal against channel 2, the communication process of channel 2 was found to be disrupted, as observed from remote estimator. However, when the attack was launched on channel 2, it disturbed the adjacent channels, which were channel 1 and channel 3. The resulting statistic extracted for this phase are shown in Figure 4. Moreover, the channel 2 attack disturbed both the adjacent channels (1 & 3), because it was in the middle. The statistics are also seen for channel 3 under a jamming attack. As in the previous example, the high frequency signal is generated in the transmission channel of the edge node 3 (channel 3). The communication of channel 3 was found to be disrupted at remote locations, because all the signals were disturbed in the transmission medium. After that they were unable to reach the destination location. Likewise, the RRT of adjacent channels were also found to be disturbed by means of inconsistent communication. The results statistics of channel 3 are shown in Figure 5 in the graphical form.

**Figure 6:**
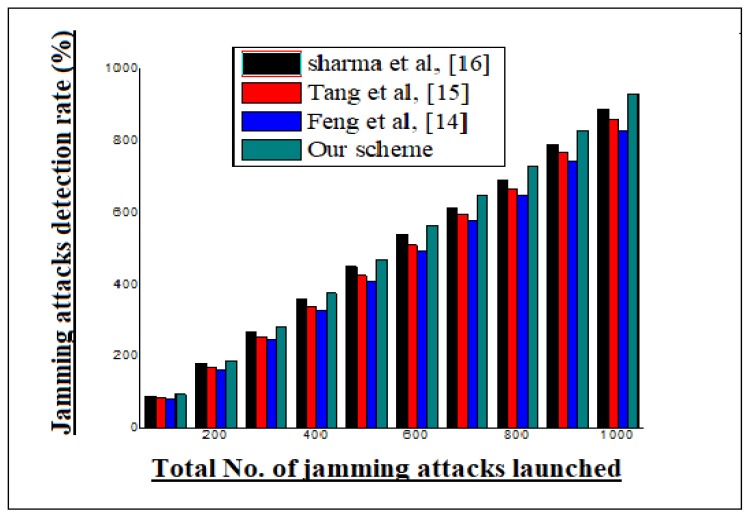
Brief statistical analysis of our scheme and its rival schemes by means of detecting attacks.

Our proposed scheme is also compared with its rival schemes on the basis of the accurate detection of a jamming attack, as shown in the channel-wise detection of jamming attacks. Our proposed model is quite efficient at identifying the affected channel by means of communication disruption from the deploying area. However, the network serviceability is verified by means of additional transmission channel from the deployed area of the WSNs. The results extracted during simulation were exceptional. This is shown for the jamming attacks statistics in each channel. However, the literature was considered and used as a comparison to assess the results of our scheme. The overall statistical analysis of our scheme in the presence of its competitor schemes is shown in Figure 6.

The proposed scheme is compared with its rival schemes on the basis of jamming attacks detection and network performance evaluation metrics, such as end to end delay and throughput.

The existing techniques only verify the detection of jamming attacks. However, in most cases, they did not consider network performance, which is an essential part of the WSNs. Therefore, we have compared our proposed scheme on the basis of jamming attacks with competitor schemes to verify its effectiveness against jamming attacks. Furthermore, we also consider the reliability metrics such as end to end delay and throughout to the network to evaluate the performance of our scheme. The results obtained during simulation of the aforementioned metrics was quite remarkable. Moreover, our scheme is not only efficient against jamming attacks, as stated earlier in the paper, but it also has very effective network performance. The statistical analysis of results are shown in the subsequent graphs.

After deep analysis of jamming attacks detection, the network performance was seen for end to end delay and throughput. Because the wireless network has limited resources, efficient utilization is the key factor to achieving results that are 100% successful.

**Figure 7:**
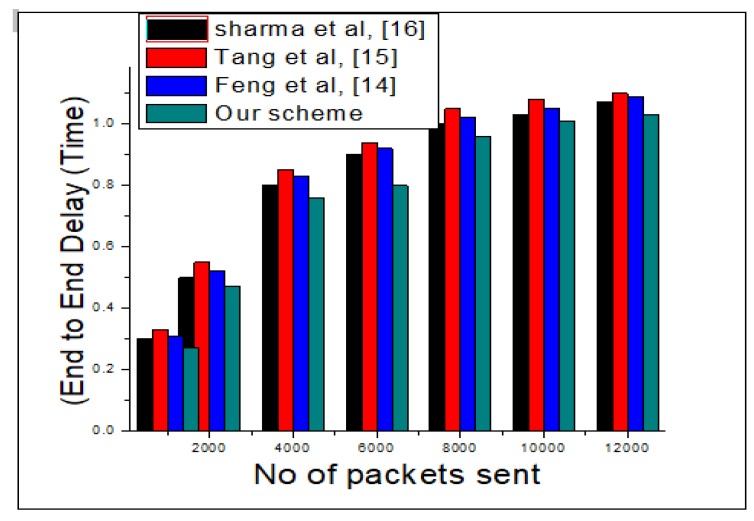
End to End delay brief statistical analysis of our scheme and rival schemes.

End to end delay is the amount of time a message takes to travel from source to destination. The statistic for this is shown in Figure 7.

**Figure 8:**
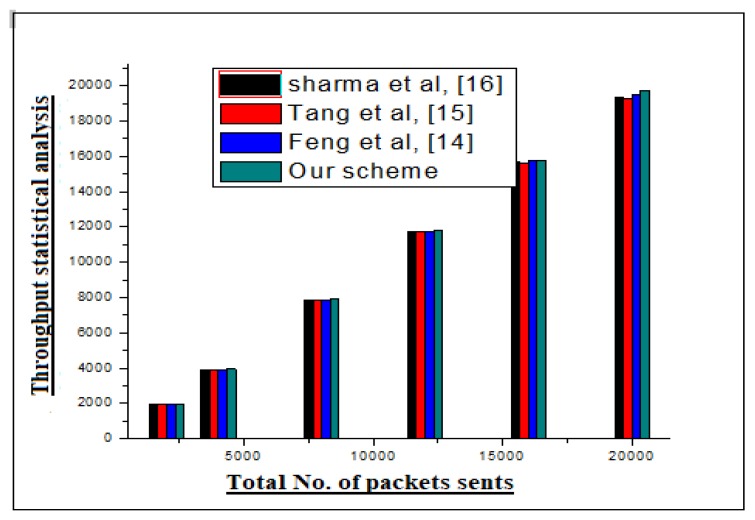
Through-put statistical analysis of our scheme and rival schemes.

Likewise, throughput is how much traffic a channel can accommodate at a time. For, this the statistics of our scheme as a whole (3 channels) are shown in Figure 8.

## 5. Conclusions and Future Work

Wireless networks perform exceptionally well, if efficiently utilized and proper security measures are taken in the deployed area. There are many security threats to these networks, which destroy their performance and utilization. Jamming attack is one the most often used damaging attacks to these networks. These attacks jam all network traffic by means of the same frequency transmission. The literature describes various schemes, but they all have limitations at some stages. Therefore, an efficient scheme was needed to address the aforementioned issue of jamming attacks. In this research, we proposed a three edge nodes scheme. Each edge node has a different transmission frequency channel in the same bandwidth. Moreover, RTT time estimation at both ends (source and destination) in a combination with different frequency channels enable us to identify a jamming attack on the network. Additionally, the proposed scheme also verifies the network serviceability at a deployed location by means of different transmission channels. Moreover, our proposed model has the provision to be applied in the operational environment of tracking control mobile robots to detect and identify jamming attacks in the form of DoS attacks, if they share their collected information via edge nodes in a deployed network. The results statistics observed for this scheme were quite attractive against jamming attacks. Similarly, the network reliability metrics of end to end delay and throughput were also observed, and were found to be quite consistent compared to competitor schemes. In the future, we are looking to extend this work for heterogeneous networks, which should be based on IoT devices.

## Figures and Tables

**Table 1:** Limitation overview of existing literature to prevent jamming attacks in Wireless Sensor Networks (WSNs).

Scheme Name	*Operation/System Specific*	*Complex Implementation & High Maintenance Cost*	*Network Overhead*	*Effectiveness against Routing Attacks*
Zhang et al. [13]	Homogeneous system	Complex to implement in real environment	High network overhead	Yes, in homogeneous networks
Feng et al. [14]	Not specific to the system	Complex to implement and maintain in real WSNs infrastructure	High	Yes
Tang et al. [15]	Yes	Normal to implement & maintain	Minimum for specific system such as Mobile robots	Yes
Sharma et al. [16]	Suitable for all systems	Complex to maintain in real environment	High network overhead, due to behavior based authentication	Not very effective in real environment to maintain high security standard, due to external interferences
Ma et al. [18]	Designed for specific for USVS	Designed for WSNs environment	Normal network overhead	Maintenance of high security standard is still challenging.

**Table 2:** Parameters used in proposed scheme implementation.

Name of Parameter	Values of Parameters
Simulation Environment	850 × 850
Simulation Tool	OMNeT++
Wireless nodes $D_{l}$	100, 200, 500, 1000
Infrastructure of the Network topology	Random Deployment
Jamming attacks detection	Check for individual channel and rival schemes
Channel categorization	Three different transmission channels
RTT Threshold value	15 μs
Idle power consumption	1.2 μW
Type of attacks	Jamming attacks
Channel Delay	2 μs
Initial hop count of sensor nodes	0
Edge nodes	3
Initial Energy of sensor nodes $E_{i}$	25,000 mAh
Bandwidth	10 Mbps
Channel Bandwidth	3 Mbps
Consumed energy during transmission of a packet ($T_{x})$	75.6 mW
Energy consumption during Sleep mode	0.7 μW
residual energy ($E_{r}$)	Total energy of ($E_{i}$)—Consumed energy of ($E_{c}$)
Transmission range	120 M
Network Traffic type	UDP and CBR
Size of Packet	128 Kbps
